# Membrane Access and Orbital Localization Govern ABC Transporter Substrate Recognition

**DOI:** 10.3390/molecules31122084

**Published:** 2026-06-13

**Authors:** Saad Harrizi, Imane Nait Irahal, Kaouthar El Birgui, Mostafa Kabine

**Affiliations:** 1 Laboratoire Santé, Environnement et Biotechnologie (LSEB), Faculté Des Sciences Ain Chock, Université Hassan II de Casablanca, Casablanca 20100, Morocco; imane.naitirahal@univh2c.ma (I.N.I.); mostafa.kabine@univh2c.ma (M.K.); 2 Laboratory of Chemistry and Biochemistry, Nutrition, Environment and Health, Faculty of Medicine and Pharmacy of Casablanca, Université Hassan II de Casablanca, Rue Tarik Ibnou Ziad, Casablanca 20250, Morocco; kaouthar.elbirgui-etu@etu.univh2c.ma; 3 Laboratory of Ecology and Environment, Faculty of Sciences Ben M’Sick, Université Hassan II de Casablanca, Avenue Cdt Driss El Harti, Casablanca 20670, Morocco

**Keywords:** Pdr5p, ABC transporters, multidrug resistance, machine learning, quantum chemical descriptors

## Abstract

The ATP-binding cassette transport protein Pdr5p is known to play a role in multidrug resistance in *Saccharomyces cerevisiae* by effluxing structurally diverse xenobiotics; yet the physicochemical determinants of substrate recognition remain poorly defined. To address this, density functional theory (DFT) calculations at the B3LYP-D3BJ/def2-SVP level were combined with machine learning to derive a predictive model of substrate recognition using a curated dataset of 66 compounds spanning 9 functional categories. A hybrid support vector machine (SVM) classifier achieved 96.3% accuracy (95% CI: 81.0–99.9%, Clopper–Pearson exact) in discriminating substrates from non-substrates under leave-one-out cross-validation. Feature importance analysis identified lipophilicity (LogP, F-score = 37.5) as the dominant descriptor, suggesting that membrane partitioning constitutes the initial recognition step. The HOMO–LUMO gap contributed secondarily (F-score = 12.4). Substrates were further distinguished by high frontier orbital focalization, with frontier orbital spread of 1.8–2.6%, compared to 4.18–7.22% for non-substrates. Notably, a model trained exclusively on Pdr5p data achieved 87% prediction accuracy when applied without retraining to the human P-glycoprotein (ABCB1) dataset, suggesting conserved physicochemical principles of substrate recognition across evolutionarily distant ABC transporters. These findings provide a quantum chemical framework for understanding and potentially predicting MDR transporter substrate specificity.

## 1. Introduction

Multidrug resistance (MDR) is a major impediment in the treatment of fungal infections and cancer, thus contributing to increased mortality rates worldwide [1,2]. In the model organism *Saccharomyces cerevisiae*, the pleiotropic drug resistance (PDR) network, mainly mediated by the transcription factors Pdr1p and Pdr3p, controls the expression of transporters that actively extrude a wide range of cytotoxic compounds [3,4]. Among these transporters, Pdr5p, the prototype of the ATP-binding cassette (ABC) family, confers resistance to antifungal agents, chemotherapeutic drugs, antibiotics, and other xenobiotics [5,6].

Pdr5p is not only an essential component of the pleiotropic drug response in *S. cerevisiae* but also serves as a valuable model for understanding multidrug resistance in humans. Indeed, Pdr5p shares significant structural and functional similarity with human ABC transporters, including ABCB1 (P-glycoprotein) and ABCG2 (BCRP), both of which are implicated in chemotherapy failure and pharmacokinetic variability [7,8]. Recent cryo-electron microscopy studies have elucidated the three-dimensional structure of Pdr5p, revealing a complex transmembrane topology with several potential substrate-binding sites, thereby providing a structural rationale for the broad polyspecificity of this transporter [9]. However, despite these advances, the physicochemical basis of substrate versus non-substrate discrimination is still not well understood.

In the past, substrates of the pleiotropic drug transporters, including Pdr5p, have been identified using experimental assays such as drug accumulation, growth inhibition, and ATPase stimulation [5,10]. Although these studies have identified a substantial number of substrates, these methods have failed to provide useful insights into the physicochemical basis of substrate transport. Early structure–activity relationship (SAR) studies suggested that hydrophobicity and the presence of nitrogen-containing heterocyclic scaffolds are important features of substrate recognition [11].

Quantum chemical approaches, particularly density functional theory (DFT), provide electronic descriptors, numerical parameters encoding the energy and spatial distribution of electrons in a molecule, which can be used to support transporter-substrate interaction models [12,13]. Within this framework, frontier molecular orbital (FMO) theory correlates chemical reactivity and interaction potential with the energy and spatial distribution of the highest occupied molecular orbital (HOMO) and lowest unoccupied molecular orbital (LUMO), respectively [14,15]. While the HOMO–LUMO energy gap is used as a general index of chemical reactivity, molecular electrostatic potential (MEP) surfaces can be used to determine charge distributions that are important in noncovalent interactions. Despite their widespread use in drug discovery and medicinal chemistry, electronic descriptors have rarely been integrated into predictive models of ABC transporter substrate recognition; existing ML approaches have relied predominantly on empirical physicochemical descriptors [16,17,18,19]. However, quantitative and predictive models capable of rationalizing substrate selectivity remained scarce. Indeed, whether membrane partitioning alone governs substrate selection, or whether direct protein-substrate electronic interactions play an equally decisive role, remains a matter of debate [8,20].

Meanwhile, machine learning techniques have revolutionized predictive modeling in pharmacology by uncovering non-linear relationships in high-dimensional descriptor spaces. In particular, support vector machines, random forests, and deep learning approaches have shown promise in predicting substrates of human efflux transporters, including P-glycoprotein and BCRP [17,18]. A major outstanding question is whether substrate recognition rules in ABC fungal transporters are species-specific or whether they reflect shared physicochemical constraints conserved across eukaryotic ABC transporters [19,21]. Resolving this question is critical to validating the use of yeast ABC transporters as mechanistically faithful models of human multidrug resistance [8,20]. We hypothesized that quantum chemical descriptors derived from frontier molecular orbital theory, combined with classical physicochemical descriptors, would improve the discrimination of Pdr5p substrates from non-substrates, and that the resulting recognition rules would be transferable to human ABC transporters without retraining.

In this study, we aimed to determine whether quantum chemical descriptors can discriminate between Pdr5p substrates and non-substrates, identify which physicochemical or electronic properties are most important in substrate recognition, and establish whether these recognition rules are conserved across species. We performed DFT calculations on 61 compounds that are established substrates and non-substrates of Pdr5p, machine learning modeling of substrate recognition, and molecular electrostatic potential analysis on selected compounds. Our results revealed that lipophilicity is the primary filter in ABC transporter substrate recognition, while electronic descriptors play a secondary role in focalizing recognition. Most importantly, we demonstrate that recognition rules are transferable to human P-glycoprotein without retraining, suggesting that shared physical principles govern ABC transporter-mediated efflux across evolutionarily distant species.

## 2. Results

### 2.1. Quantum Chemical Landscape of Pdr5p-Associated Compounds

Geometry optimizations were successful for 61 of 66 compounds (92.4%) with five large molecules (MW > 1000 Da) omitted due to computational limitations. Of the 61 successfully optimized compounds, a training subset of 27 was used for classifier development; 34 compounds were excluded due to absence of quantitative resistance data or resistance ratios in the ambiguous 1.5–3-fold range that preclude unambiguous labeling. The resulting dataset spans a broad range of chemical diversity, with molecular weights ranging from 75.07 Da (glycine) to 924.08 Da (amphotericin B), and HOMO–LUMO gaps from 2.88 eV (daunorubicin) to 7.32 eV (glucose).

Electronic descriptors show distinct category-dependent trends (Table 1). Chemotherapeutic agents have the smallest mean HOMO–LUMO gap (3.70 ± 1.07 eV), consistent with large conjugated π-systems and increased electronic reactivity. In contrast, non-substrate controls have significantly larger gaps (5.99 ± 1.17 eV), indicating chemical stability and decreased interaction propensity. Azoles, antifungal compounds, have medium-range gaps (4.73 ± 0.59 eV), whereas membrane stress compounds have medium-range gaps (4.35 ± 0.94 eV).

Substrate status and resistance magnitude for all compounds were determined based on consolidated experimental evidence from multiple research groups and different assay types.

### 2.2. Conformational Energy Landscapes Reveal Contrasting Flexibility Profiles

Fluconazole was selected as the most clinically relevant Pdr5p substrate and glucose as the canonical non-substrate control employed throughout the Pdr5p literature, providing a substrate/non-substrate contrast that is both biologically meaningful and internally consistent with the full analysis. These two compounds are not intended as a general survey of conformational diversity across the dataset but serve as mechanistically illustrative examples supporting the hierarchical recognition model.

**Figure 1:**
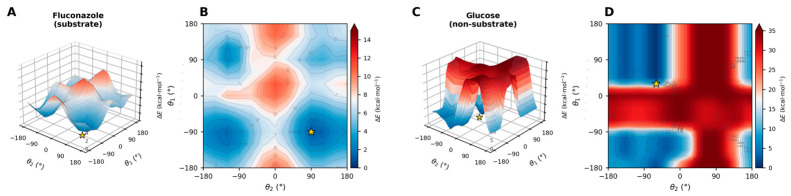
Conformational energy landscapes of fluconazole (substrate, panels (**A**,**B**)) and glucose (non-substrate, panels (**C**,**D**)). (**A**,**C**) Three-dimensional relaxed potential energy surfaces as a function of dihedral angles θ_1_ and θ_2_. (**B**,**D**) Two-dimensional contour maps of the same surfaces with 1 kcal·mol^−1^ contour intervals. Energies are referenced to the global minimum and clipped at 15 kcal·mol^−1^ (fluconazole) and 35 kcal·mol^−1^ (glucose) for visualization. Stars indicate global minima at (θ_1_ = −90°, θ_2_ = 90°) and (θ_1_ = 45°, θ_2_ = −60°) respectively.

2D relaxed potential energy surface scans were performed on fluconazole and glucose as representative substrate and non-substrate molecules, respectively, to probe their conformational flexibility. The fluconazole molecule revealed a relatively smooth conformational map with a total energy window of 13.9 kcal·mol^−1^ after correction of three SCF convergence failures by cubic interpolation (Figure 1). The global minimum was found at θ_1_ = −90° and θ_2_ = 90°, signifying a perpendicular arrangement of the two triazole rings with respect to the central linker. The local minimum around θ_1_ ≈ 90° and θ_2_ ≈ −120° was found to be separated from the global minimum by barriers of around 8–10 kcal·mol^−1^. The potential energy surface revealed C_2_ symmetry around θ_1_ = 0°. The broad areas of low energy (<5 kcal·mol^−1^) covered around 40% of the conformational space around the global minimum. The moderate flexibility of fluconazole could be important for adapting to the polyspecific Pdr5p binding cavity, as compared to glucose, which is highly inflexible and features extensive intra-molecular hydrogen bonding and is therefore incompatible with the hydrophobic transport environment. Importantly, this conformational contrast is consistent with the lipophilicity-gated recognition model: glucose’s rigid, hydroxyl-rich scaffold not only fails the LogP filter but also lacks the conformational adaptability required to access the transporter binding pocket.

By contrast, the conformational landscape of glucose was considerably more restricted. The combined bidirectional surface had a width of 145.3 kcal·mol^−1^ (Figure 1C,D), although visualization cut off at 35 kcal·mol^−1^ showed a very narrow valley at low energy, centered around the global minimum (θ_1_ = 45°, θ_2_ = −60°). The accessible conformations (ΔE < 10 kcal·mol^−1^) represented ~25% of the grid, showing a much more rigid structure than fluconazole. The high energy barriers reflect the constrained rotation of hydroxyl groups in the sugar ring and the strong intramolecular hydrogen-bonding network typical of carbohydrate structures.

**Figure 2:**
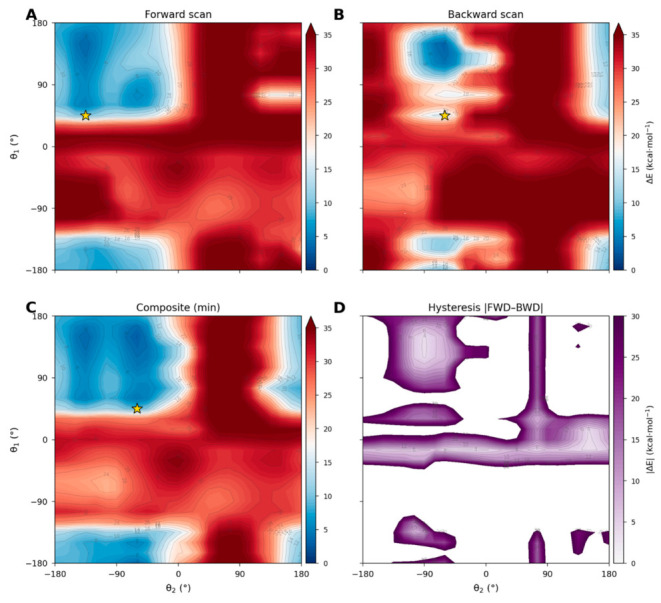
Bidirectional conformational scan analysis for glucose. (**A**) Forward dihedral scan (−180° → 180°). (**B**) Backward dihedral scan (180° → −180°). (**C**) Composite potential energy surface obtained by selecting the lower-energy structure at each grid point. (**D**) Hysteresis map showing the absolute energy difference between forward and backward scans (|ΔE_FWD − ΔE_BWD|). Energies are referenced to the global minimum and clipped at 35 kcal·mol^−1^. The stars indicate the global energy minimum in each conformational landscape.

The bidirectional scanning results show significant path dependence in the glucose PES (Figure 2). Of the 625 grid points for which data were available in both directions, the forward scan yielded the lowest energy at 364 points (58.2%), the reverse scan at 251 points (40.2%), and both directions converged to equal energies at the remaining 10 points. The largest discrepancies (>25 kcal·mol^−1^) occurred in the region around θ_1_ = 0–30°, where optimizations converged to different local minima depending on the scanning direction. This path dependence is a methodological consequence of the glucose conformational landscape and confirms that bidirectional scanning is necessary to construct a reliable composite PES for this type of flexible polar molecule; it does not affect the substrate classification results reported elsewhere.

### 2.3. Frontier Orbital Focalization Distinguishes Substrates from Non-Substrates

**Figure 3:**
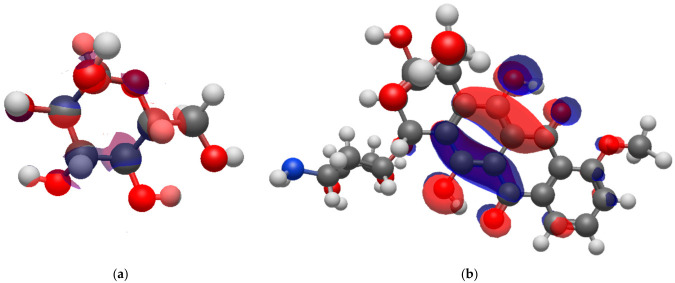
Frontier orbital focalization distinguishes transported substrates from non-substrates. HOMO isosurfaces (rendered at ±0.02 a.u.) are shown for two representative compounds: (**a**) glucose, a non-substrate, and (**b**) doxorubicin, a Pdr5p substrate. Glucose exhibits diffuse, spatially distributed frontier orbitals spread across multiple hydroxyl groups (HOMO spread = 4.18%), whereas doxorubicin displays pronounced orbital focalization localized on its aromatic core (HOMO spread = 1.8%). This contrast illustrates that frontier orbital focalization acts as a categorical electronic signature of substrate recognition, rather than a quantitative predictor of transport efficiency. The color scale represents relative conformational energy in kcal·mol^−1^, from blue (low energy) to red (high energy).

Analysis of the frontier orbital spatial distribution for substrates and non-substrates indicates that there is a clear distinction in the frontier orbital distribution for substrates compared to non-substrate molecules (Table 2). Substrates of Pdr5p exhibit compact, spatially localized frontier orbitals. The values of HOMO spread for different categories of substrates range from 1.79 to 2.56%. In contrast, non-substrate molecules have highly distributed frontier orbitals with a high HOMO spread of 4.18%, whereas the LUMO orbitals show high values of orbital delocalization at 7.22% for glucose molecules. Among chemotherapeutic agents, there is high orbital localization with a low HOMO spread of 1.83%, consistent with their planar aromatic ring structure. In the anthracycline class of chemotherapeutics, there is high similarity in frontier orbital distribution for both doxorubicin and daunorubicin with values of 1.74% and 1.78%, respectively. In the case of glucose molecules, there is high orbital delocalization with a high value of 7.22% for the LUMO orbitals. The frontier orbital distribution for a substrate and a non-substrate molecule is represented in Figure 3.

### 2.4. Lipophilicity Is the Dominant Predictor of Pdr5p Substrate Recognition

To determine which molecular descriptors would be the most effective at distinguishing between substrates and nonsubstrates, feature selection was employed on the training set. The results highlighted the importance of the lipophilicity descriptor, as it produced the highest F-score at 37.55, which far exceeds the score produced by the HOMO–LUMO gap at 12.38. The five most important features were found to be the LogP descriptor, the hydrogen-bonding capacity corrected for molecular weight, the polar surface area corrected for molecular weight, the interaction term Gap × LogP, and the HOMO–LUMO gap.

**Figure 4:**
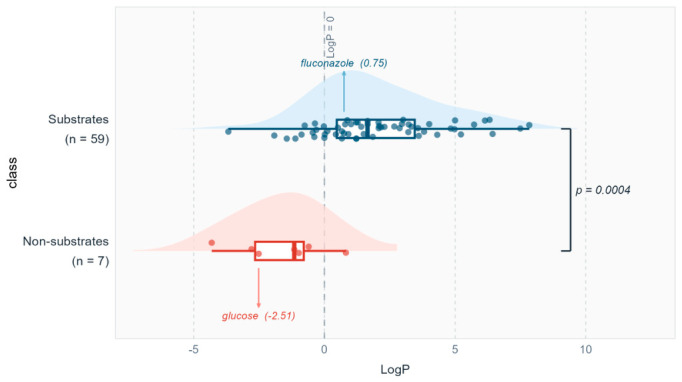
LogP distributions of Pdr5p substrates and non-substrates across the full annotated dataset. Box plots show the interquartile range; whiskers extend to 1.5 × IQR; individual compounds are overlaid as dots. LogP values were computed using the Wildman–Crippen model as implemented in RDKit [22,23]. The dashed vertical line marks LogP = 0, the empirical membrane-access threshold. Substrates (n = 59) are distributed predominantly above this threshold (median LogP = 1.90), whereas non-substrates (n = 7) cluster predominantly below this threshold (median LogP = −1.03). Two-sided Mann–Whitney U test: *p* < 0.0001. Caffeine (LogP  =  0.06) is the single non-substrate above LogP  =  0 (marked). Nine substrate compounds with LogP  <  0 represent oxidative stress metabolites and hydrophilic chemotherapeutic agents whose efflux is driven by conjugation-dependent mechanisms. Colors distinguish substrate (blue) from non-substrate (red) classes.

**Figure 5:**
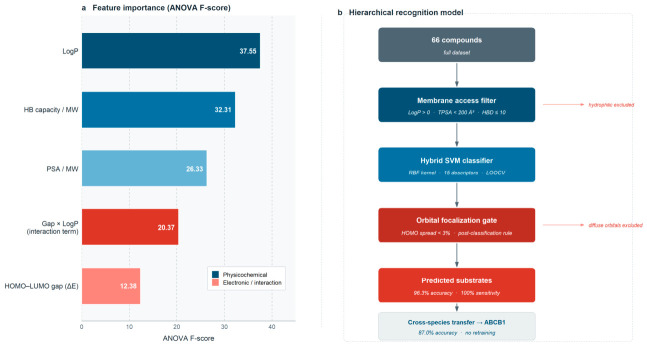
Feature importance and the hierarchical recognition model for Pdr5p substrate selection. (**a**): ANOVA F-scores of the five most discriminative descriptors identified by feature selection. LogP dominates with an F-score of 37.55, nearly three times that of the HOMO–LUMO gap (12.38). The interaction term Gap × LogP ranks fourth, indicating partial synergy between electronic reactivity and membrane partitioning. (**b**): hierarchical decision framework derived from these rankings. Lipophilicity (LogP) acts as a membrane-access gate; compounds below the threshold are excluded before any electronic evaluation. Among membrane-accessible compounds, frontier orbital focalization (spread < 3%) provides secondary discrimination. This ordered framework illustrates that substrate recognition operates through sequential physical constraints rather than additive descriptor contributions. The varying color shades represent the continuous energy gradient as described in the color bar.

This ranking of the descriptors emphasizes the importance of the lipophilicity descriptor as a major factor for substrate recognition, which supports the idea of the membrane access first model for substrate-transporter interaction. The LogP distributions are substantially separated between substrate and non-substrate classes (Mann–Whitney U  =  355, *p*  =  1.80 × 10^−4^; rank-biserial r  =  0.878; substrate median  =  1.78, non-substrate median  =  −0.97). The majority of non-substrates (86%, 6/7) cluster below LogP  =  0, with one exception: caffeine (LogP  =  0.06), a borderline compound. Conversely, 9 substrate compounds (17%) exhibit LogP < 0, predominantly oxidative stress metabolites and hydrophilic chemotherapeutic agents whose efflux is driven by conjugation-dependent mechanisms. Despite these exceptions, the overall LogP separation is strong (CLES  =  0.939), and the substrate distribution is centered above 1.5 (Figure 4), directly visualizing the membrane-access threshold that underlies this discriminatory power. The electronic descriptors also played a role as a secondary discriminatory factor, which supports the idea of a hierarchical model for substrate recognition. The resulting hierarchy of decision logic is schematically represented in Figure 5. To quantify the independent contribution of quantum chemical descriptors, five SVM models were evaluated on the same 61-compound dataset using leave-one-out cross-validation: a single-descriptor LogP baseline (M0), classical physicochemical descriptors (M1, 6 features), quantum descriptors alone (M2, 6 features), and two combined models (M3: 12 features; M4: 15 features including the interaction term Gap  ×  LogP and MW-normalised polar descriptors). A single LogP descriptor achieved a balanced accuracy of 90.7% and MCC  =  0.579, confirming membrane partitioning as the dominant discriminator. Classical descriptors alone (M1) reached 77.4% balanced accuracy (MCC  =  0.415), while quantum descriptors alone (M2) achieved 73.0% (MCC  =  0.396; permutation *p*  =  0.032), demonstrating independent but secondary discriminatory power. The full 15-descriptor model (M4) achieved the highest performance: 84.8% balanced accuracy, MCC  =  0.745, and 95.1% overall accuracy (permutation *p*  =  0.002). The MCC improvement from classical-only (M1: 0.415) to the full model (M4: 0.745; ΔMCC  =  0.330) demonstrates that quantum chemical descriptors—particularly the HOMO–LUMO gap and its interaction with LogP—provide substantial complementary discriminatory power (Table 3).

### 2.5. Hybrid Machine Learning Model Achieves High Accuracy with Perfect Substrate Sensitivity

Among the classifiers, the best baseline performance was observed with the SVM classifier using the RBF kernel, which showed 92.6% accuracy under leave-one-out cross-validation (Table 4). Close performance was observed with logistic regression and random forest classifiers, which showed 88.9% accuracy. The k-nearest neighbors classifier achieved comparable accuracy to SVM (92.6%) but lower specificity (57.1% versus 71.4%), reflecting its reduced ability to correctly reject non-substrates.

All classifiers showed 100% sensitivity, correctly classifying all experimentally known substrates, while specificity ranged from 57.1% to 71.4%. Physically motivated constraints, which are used after classification, showed significant improvements in specificity. With these constraints, the classifier showed 96.3% accuracy (95% CI: 81.0–99.9%), 85.7% specificity (balanced accuracy: 92.8%), and an AUC-ROC score of 0.93. Only one compound, caffeine, was classified as a substrate. It should be noted, however, that caffeine showed borderline experimental behavior, with approximately 1.2-fold resistance, placing it at the boundary of classification.

### 2.6. Weak Correlation Between Electronic Reactivity and Resistance Magnitude

Despite clear separation between substrates and non-substrates, correlations between individual electronic parameters and resistance magnitude were found to be poor. The HOMO–LUMO gap showed minimal correlation with resistance ratios (Pearson r = −0.11, *p* = 0.63). This seeming contradiction is exemplified by fluconazole, which has one of the largest HOMO–LUMO gaps of antifungal drugs (6.06 eV) and yet results in extremely high resistance values (~638-fold), whereas doxorubicin, having a much smaller gap (2.91 eV), results in relatively lower resistance values (~4-fold).

It should be noted that resistance magnitude does not necessarily reflect transport efficiency directly. Resistance phenotypes can arise from mechanisms other than active efflux, including competitive inhibition and indirect growth effects, as demonstrated by studies employing orthogonal experimental systems [10,24].

### 2.7. Cross-Species Validation Reveals Conserved ABC Transporter Recognition Rules

To assess whether the substrate recognition rules learned by the model on the Pdr5p dataset are species-specific or evolutionarily conserved, the model was used to predict the substrates of the human P-glycoprotein (human ABCB1). It is important to note that this was done without any model retraining, fine-tuning, or parameter adjustment.

The model was able to achieve an accuracy of 87.0%, with a sensitivity of 100%, specificity of 60.0%, F1-score of 0.912, and Matthews correlation coefficient (MCC) of 0.709. Lipophilicity remained the primary discriminating feature, consistent with the hierarchical recognition framework established in the Pdr5p model.

The most interesting finding was that the performance of the transferred model, which was trained on the Pdr5p dataset, was comparable to that of models trained on the ABCB1 dataset, despite the fact that the datasets were completely independent of each other. These results provide proof-of-concept evidence that ABC transporter substrate recognition may be subject to evolutionarily conserved physicochemical constraints. However, the 60% specificity on ABCB1—while within the range reported for purpose-built ABCB1 classifiers trained on human data (55–75% [17,18])—reflects the inherent difficulty of cross-species transfer and the limited size of the validation set. Validation on additional transporters such as ABCG2 and fungal Cdr1p is required before broader generalization claims can be made.

### 2.8. Integrated View of Substrate Recognition

**Figure 6:**
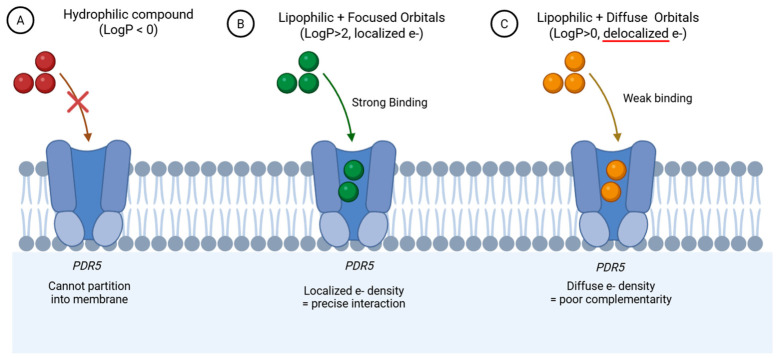
Membrane-gated electronic recognition model of Pdr5p substrate selection. Conceptual model illustrating how lipophilicity and electronic structure jointly govern substrate recognition. (**A**) Hydrophilic compounds fail to partition into the lipid bilayer and are excluded from recognition. (**B**) Lipophilic compounds with focalized frontier orbitals efficiently interact with the transporter binding cavity, enabling recognition and transport. (**C**) Lipophilic compounds with diffuse electronic distributions exhibit suboptimal electronic complementarity, resulting in weak or absent recognition. This model integrates membrane partitioning and electronic focalization into a unified framework for ABC transporter polyspecificity.

Collectively, these findings support a model of hierarchical compound recognition wherein lipophilicity is a determinant of membrane access, and electronic features such as frontier orbital focalization are secondary determinants of compound recognition within accessible chemical space. The magnitude of resistance and transport efficiency are also revealed as related but distinct phenomena influenced by additional determinants such as multiplicity of binding site recognition and assay context. This model reconciles the disconnect between electronic reactivity and resistance magnitude and provides insight into how structurally diverse compounds are recognized by a single polyspecific ABC transporter. These constraints are accommodated within a membrane-gated electronic recognition model as shown in Figure 6.

## 3. Discussion

This study confirms our hypothesis that combining quantum chemical descriptors derived from frontier molecular orbital theory with classical physicochemical descriptors provides superior discriminatory power for ABC transporter substrate recognition, and that the resulting recognition rules are transferable to human ABC transporters without retraining. The central finding is that lipophilicity dominates substrate recognition for Pdr5p, exceeding the predictive power of quantum chemical electronic descriptors even when derived from high-level DFT calculations [12,13]. While the HOMO–LUMO gap is frequently used as a proxy for molecular reactivity [14], our feature importance analysis shows that LogP ranked approximately threefold higher than the HOMO–LUMO gap by ANOVA F-score within this dataset—a relative ranking consistent with the mechanistic primacy of membrane partitioning.

This result is consistent with the mechanistic framework of ABC transporter function, in which substrates are captured from within the lipid bilayer rather than directly from the aqueous cytoplasm [7,9]. Compounds must therefore partition into the membrane before interacting with the transporter binding cavity. Lipophilicity acts as a primary membrane-access filter that defines the chemical space available for recognition [25], extending and quantitatively validating earlier SAR observations that identified hydrophobicity as a general feature of Pdr5p substrates [11].

The successful transfer of the Pdr5p-trained model to human P-glycoprotein without retraining demonstrates that this physicochemical constraint is evolutionarily conserved across kingdoms of life [7,26]. Rather than representing a transporter-specific heuristic, lipophilicity appears to reflect a fundamental physical requirement imposed by the lipid bilayer, supporting a membrane-access–first model of substrate recognition [8,20].

Existing machine learning models for ABC transporter substrate prediction have relied predominantly on empirical physicochemical descriptors, without incorporating quantum chemical descriptors capturing electronic structure [17,18]; to the best of our knowledge, zero-shot cross-species transfer without retraining has not been reported in this context. The present framework advances the field by demonstrating that frontier orbital focalization constitutes an independent discriminatory signal inaccessible to purely empirical approaches, and by validating learned decision boundaries on an independent cross-species dataset without retraining.

Beyond membrane accessibility, frontier orbital focalization emerges as a categorical electronic feature distinguishing substrates from non-substrates [14,15]. Transported compounds consistently exhibit compact, spatially localized frontier orbitals, whereas non-substrates display diffuse electronic distributions. Because orbital focalization reflects the spatial organization of electron density rather than global electronic reactivity, it likely facilitates productive non-covalent interactions with residues lining the transporter binding cavity [9]. Notably, orbital focalization does not correlate with resistance magnitude within the substrate class. Instead, it appears to function as a recognition gate, distinguishing molecules compatible with the transporter binding environment from those that are electronically incompatible [24]. This distinction suggests that substrate recognition and transport efficiency are governed by partially independent constraints.

The weak correlation between electronic reactivity and resistance magnitude further highlights this principle. The fluconazole paradox, high resistance despite a relatively large HOMO–LUMO gap, illustrates that intrinsic molecular reactivity does not determine efflux efficiency [5,10]. Cryo-EM structures of Pdr5p reveal multiple partially overlapping binding cavities [9], consistent with a multi-site recognition architecture in which resistance magnitude reflects site occupancy and binding kinetics rather than electronic reactivity alone [24].

The hybrid classification framework achieved 96.3% accuracy with perfect substrate sensitivity, outperforming previously reported ABC transporter predictors [17,18]. It must be emphasized that this accuracy is a point estimate on 27 samples (26/27 correctly classified); the 95% Clopper–Pearson confidence interval is [81.0%, 99.9%], reflecting the small sample size. The result should be interpreted as proof-of-concept rather than a validated generalization bound, and balanced accuracy (92.8%) and MCC (0.904) provide class-imbalance-robust performance estimates. The single misclassified compound, caffeine, represents a biologically ambiguous case with borderline resistance behavior, illustrating that substrate recognition likely exists along a continuum rather than as a strict binary state [24]. Several limitations must be acknowledged: the training set comprises 27 compounds, which constrains statistical power and produces an unfavorable feature-to-sample ratio; binary classification simplifies an inherently continuous transport process; and DFT calculations on single conformers in implicit solvent do not capture membrane-environment polarization effects or conformational dynamics relevant to binding [8,20,21].

## 4. Materials and Methods

### 4.1. Dataset Compilation

A curated dataset of 66 compounds was assembled from previously published studies reporting experimental interactions with the *Saccharomyces cerevisiae* ABC transporter Pdr5p and associated multidrug efflux systems [5,6,10,27]. The compounds were classified into nine functional groups based on primary pharmacological activity or biochemical role: antifungals (14), oxidative stress modulators (12), negative controls (7), membrane stress inducers (6), antibiotics (5), chemotherapeutic agents (5), natural products (5), steroids (4), and agricultural fungicides (3). This dataset represents, to the best of our knowledge, the most comprehensive collection of compounds with reliable experimental Pdr5p transport annotations currently available in the literature.

Substrate status was based on the consolidated body of experimental evidence from existing literature regarding resistance assays, intracellular accumulation, ATPase, and transport experiments [5,6,10,24]. Non-substrate status was assigned to endogenous metabolites, such as glucose and amino acids, and to compounds previously reported to have minimal or no interaction with Pdr5p. For model training, compounds with resistance ratios in the range of 1.5- to 3-fold were excluded to minimize label ambiguity. The four excluded compounds were: caffeine (1.2-fold), cycloheximide (2.1-fold), brefeldin A (1.8-fold), and cis-diaminedichloroplatinum (2.4-fold). This resulted in a training subset of 27 compounds with unambiguous classification labels. Full compound metadata, including compound name, functional category, substrate/non-substrate label, resistance magnitude, assay type(s), evidence quality rating, inclusion flags for each analysis (DFT, ML, orbital spread), LogP value, and the primary literature references, are provided in Supplementary Table S1. Full compound metadata, including assay types, resistance values, evidence quality, and primary references, are provided in Supplementary Table S1.

### 4.2. Three-Dimensional Structure Generation

Molecular structures were retrieved from the PubChem database [28] or directly generated from SMILES strings where necessary. Three-dimensional coordinates were obtained using the RDKit library (version 2023.03) [22] with the ETKDG distance geometry algorithm [29]. For each compound, 50 conformers were generated and optimized using the MMFF94 force field [30]. The conformer with the lowest energy was selected for subsequent quantum chemical calculations. Optimized geometries were exported in XYZ format.

### 4.3. Density Functional Theory Calculations

All quantum chemical calculations were performed using the ORCA program package (version 5.0.4) *(MPI für Kohlenforschung, Mülheim an der Ruhr, Germany)* [31]. Geometry optimizations were carried out using the B3LYP-D3BJ method with the def2-SVP basis set [32,33], combining the B3LYP hybrid functional with Grimme’s D3BJ dispersion correction with Becke–Johnson damping [34]. The def2-SVP basis set was selected for its established balance between computational efficiency and accuracy for organic molecules [32]. The resolution-of-identity approximation with the def2/J auxiliary basis set [35] was employed to accelerate Coulomb integral evaluation. Geometry optimizations were performed using tight convergence criteria (TightOpt keyword), and numerical integration was carried out using a Grid5 integration grid. All calculations were performed in the gas phase on single optimized conformers. For lipophilic substrates (LogP < 1.5), which constitute the majority of the training set, gas-phase descriptors are expected to approximate the relevant membrane-phase electronic environment. For hydrophilic non-substrates such as glucose and amino acids, implicit solvation effects may be more significant; incorporation of CPCM solvent corrections for this subset represents a direction for future work [36]. To assess the adequacy of def2-SVP geometries for computing frontier orbital descriptors, three representative molecules spanning a range of molecular complexity and substrate classification were fully re-optimised at the B3LYP-D3BJ/def2-TZVP level: glycine (10 atoms, non-substrate), caffeine (24 atoms, borderline), and fluconazole (34 atoms, primary substrate). The HOMO–LUMO gap computed on the TZVP-optimised geometry (TZVP//TZVP) differed from the single-point TZVP value on the SVP geometry (TZVP//SVP) by 0.026 eV (glycine), 0.051 eV (caffeine), and 0.079 eV (fluconazole). All geometry effects fall below the standard DFT basis set convergence threshold of 0.1 eV, confirming that def2-SVP geometry optimisations are adequate for the frontier orbital descriptors used in this study.

Furthermore, to assess whether gas-phase optimizations adequately represent the electronic properties of compounds in a polar environment, SMD implicit solvation calculations (water, ε = 78.4) at the B3LYP-D3BJ/def2-TZVP level were performed on three non-substrate controls (glycine, glucose, alanine) and two representative substrates (fluconazole, caffeine). The substrate/non-substrate HOMO–LUMO gap ordering was preserved and enhanced in implicit solvent: the mean non-substrate gap increased from 6.83 eV (gas phase) to 7.23 eV (SMD water), while the substrate gap remained stable (6.06 → 6.15 eV), widening the class separation from 0.76 eV to 1.07 eV.

From the optimized geometries, the following electronic properties were computed: HOMO energy (EHOMO), LUMO energy (ELUMO), HOMO–LUMO energy difference (ΔE = ELUMO − EHOMO), total electronic energy, and dipole moment. Conceptual DFT descriptors were calculated as follows [13]:

Chemical hardness: η = (ELUMO − EHOMO)/2

Electronegativity: χ = −(EHOMO + ELUMO)/2

Electrophilicity index: ω = χ^2^/(2η)

### 4.4. Conformational Potential Energy Surface Calculations

To investigate conformational flexibility, two-dimensional (2D) relaxed potential energy surface (PES) scans were carried out using the GFN2-xTB semi-empirical method implemented in the xtb program package (version 6.5.1) *(Universität Bonn, Bonn, Germany)* [37,38] for fluconazole and glucose, representing a known substrate and non-substrate of Pdr5p, respectively. The geometries used as starting points for the 2D scans were taken from the DFT-optimized structures described in Section 4.3.

For fluconazole, the dihedral angles θ_1_ (C2–N3–C4–C5) and θ_2_ (C2–C9–C10–C11), describing the rotation of the two triazole rings relative to the central linker, were scanned over a 13 × 13 grid (169 points) from −180° to +180° with a step size of 30°. Atom numbering corresponds to that shown in Figure 1. For glucose, the dihedral angles θ_1_ (O1–C2–C3–O4) and θ_2_ (O12–C11–C9–O10), describing the orientation of the hydroxymethyl group and a key substituent, were scanned over a 25 × 25 grid (625 points) from −180° to +180° with a step size of 15°. To reduce path dependence, θ_2_ was varied in both directions and the lowest energy structure was retained at each grid point [39].

At each grid point, dihedral angles θ_1_ and θ_2_ were constrained with a harmonic force constant of 1.5 Eh rad-2, while all remaining degrees of freedom were fully relaxed using tight optimization settings [37]. Three grid points on the fluconazole PES exhibiting energies exceeding 30 kcal mol^−1^ above the surface median, resulting from SCF convergence failures, were replaced by interpolation between neighboring points using the interpolation functions of the SciPy library (version 1.11.0) [40]. PES plots were generated using the matplotlib library (version 3.7.0) [41] and visualized using the VMD program (version 1.9.4) *(University of Illinois at Urbana-Champaign, Urbana, IL, USA)* [42] and displayed as both 3D surface plots and 2D filled contour plots, with energy ranges truncated to 15 kcal mol^−1^ and 35 kcal mol^−1^ for fluconazole and glucose, respectively.

### 4.5. Molecular Electrostatic Potential and Orbital Localization Analysis

For a representative subset of 14 molecules spanning all functional categories, single-point calculations were performed at the B3LYP-D3BJ/def2-TZVP level [43] to obtain higher-quality electron density and orbital data. Cube files for the electron density and the HOMO and LUMO orbitals were generated with a grid spacing of 0.1 Bohr.

Frontier orbital localization was quantified as the percentage of grid points at which the absolute value of the orbital amplitude exceeded 0.01 a.u. This threshold was selected as it captures chemically meaningful orbital density while excluding negligible tail contributions, providing a normalized measure of the degree of spatial delocalization of the frontier orbitals [14,15]. It should be noted that this metric is not a standard localization index. A systematic sensitivity analysis at 0.005, 0.01, and 0.02 a.u. confirmed that the qualitative substrate/non-substrate separation is robust to threshold choice: glucose HOMO spread exceeds the substrate mean by 1.9×, 2.0×, and 2.1× at the three respective thresholds, with no inversions observed at any threshold (Table 5). Absolute spread values are threshold-dependent, but the biological interpretation is preserved. Note on compound subsets: different analyses in this study use partially overlapping compound sets, as detailed in Supplementary Table S1. Geometry optimizations converged for 61 of 66 compounds (five large-MW compounds excluded). Orbital spread analysis was performed on a representative subset of 14 compounds for which cube files were generated, selected to cover all functional categories while remaining computationally tractable. Cube files were generated in ORCA with an 80  ×  80  ×  80 uniform Cartesian grid; voxel spacing was automatically adjusted per molecule (0.349–0.796 Bohr) to enclose the molecular extent with approximately 4 Bohr of padding. The non-substrate  >  substrate ordering was verified to be robust to grid-box parameters: across four independent grid manipulations (10% crop, 20% crop, 20% padding extension, 2× coarser grid) × three amplitude thresholds × two orbitals, the ordering was preserved in 60/60 (100%) of pairwise comparisons (Spearman ρ  ≥  0.873 for HOMO, ρ  ≥  0.960 for LUMO, all *p*  <  5 × 10^−5^; Supplementary Table S3).

### 4.6. Physicochemical Descriptor Calculations

Complementary physicochemical descriptors were computed using the RDKit library [22]. These include molecular weight (MW), the octanol–water partition coefficient (LogP, using the Wildman–Crippen model [23]), topological polar surface area (TPSA [44]), the number of hydrogen bond donors (HBD), the number of hydrogen bond acceptors (HBA), the number of rotatable bonds, and the number of heavy atoms. To minimize size-dependent effects, the number of heavy atoms and the number of rotatable bonds were normalized by dividing by molecular weight.

### 4.7. Machine Learning Model Development and Evaluation

For model training, a subset of 27 compounds with unambiguous quantitative resistance ratios was used, as described in Section 4.1. This training set size is a recognized limitation of the study; the feature-to-sample ratio (15 features, 27 compounds) is unfavorable, and all accuracy metrics should be interpreted as point estimates with substantial uncertainty. Given the limited training set size inherent to the experimental dataset, leave-one-out cross-validation (LOOCV) was adopted as the primary validation strategy, as it provides an approximately unbiased estimate of generalization error for small datasets [45]. All features were standardized using z-score normalization prior to model training. Feature selection was performed using univariate ANOVA F-tests, retaining the 15 most discriminative features from an initial pool of 31 descriptors. The top five features by F-score were: LogP (37.55), hydrogen-bonding capacity normalized by molecular weight (32.31), polar surface area normalized by molecular weight (26.33), the interaction term Gap × LogP (20.37), and HOMO–LUMO gap (12.38).

The following classification algorithms were implemented using the scikit-learn library (version 1.3.0) [46]: logistic regression (L2 regularization, C = 0.1), random forest (200 trees, maximum depth = 3), support vector machine (RBF kernel, C = 10, gamma = scale), and k-nearest neighbors (k = 3, distance weighting). Hyperparameters for all models were selected by exhaustive grid search with LOOCV. The full search grids and optimal values were as follows: SVM (C ∈ {0.01, 0.1, 1, 10, 100}, γ ∈ {scale, auto, 0.001, 0.01, 0.1}; optimal: C = 10, γ = scale); logistic regression (C ∈ {0.001, 0.01, 0.1, 1, 10}; optimal: C = 0.1); random forest (n_estimators ∈ {50, 100, 200}, max_depth ∈ {2, 3, 5, None}; optimal: 200 trees, max_depth = 3); k-NN (k ∈ {1, 3, 5, 7}, weights ∈ {uniform, distance}; optimal: k = 3, distance weighting). The values reported in Section 4.7 correspond to the optimal configuration identified for each algorithm. Model performance was assessed using accuracy, sensitivity (true positive rate for substrates), specificity (true negative rate for non-substrates), and the area under the receiver operating characteristic curve (AUC-ROC).

### 4.8. Post-Classification Physicochemical Filtering

To increase specificity for borderline compounds, physicochemical post-classification constraints were applied to support vector machine predictions as a hard deterministic filter: any compound meeting any criterion below is reclassified as a non-substrate regardless of its SVM score. These constraints encode membrane accessibility requirements derived from established drug-likeness thresholds [25] and are entirely independent of the training labels. Critically, these rules were applied exclusively to LOOCV test-fold predictions—each compound was removed from training before its SVM score was computed—ensuring no data leakage. Compounds were predicted as non-substrates if they satisfied any of the following criteria derived from established drug-likeness thresholds:

(1) TPSA > 200 Å^2^ combined with MW > 800 Da, indicating poor membrane permeability [25];
(2) LogP < −2, indicating excessive hydrophilicity incompatible with lipid-phase access;
(3) HBD > 10, indicating extensive hydrogen bonding capacity likely to impede passive diffusion to the transporter [47].

### 4.9. Cross-Species External Validation on Human ABCB1

To assess the generalizability of the substrate recognition rules learned from Pdr5p data, the classifier trained on the yeast-derived dataset was applied without retraining, fine-tuning, or parameter modification to an independent human-derived dataset of P-glycoprotein (ABCB1) substrates and non-substrates [7,26]. Annotations for the ABCB1 dataset were compiled from published transport and inhibition studies and from the ChEMBL, DrugBank, and TransportDB databases. Overlap between the Pdr5p training set and the ABCB1 validation set was verified and confirmed to be absent. Physicochemical and quantum chemical descriptors for the ABCB1 dataset were computed using the identical pipeline described in Section 4.3 and Section 4.6. Classifier performance on the ABCB1 dataset was evaluated using accuracy, sensitivity, specificity, F1 score, and Matthews correlation coefficient [48].

## 5. Conclusions

ABC transporter substrate recognition is governed by a hierarchy of conserved physicochemical constraints. Lipophilicity functions as a membrane-imposed primary filter, defining the chemical space accessible to Pdr5p, while frontier orbital focalization provides a secondary electronic criterion that distinguishes transported compounds from those that are electronically incompatible with the binding cavity. These two constraints operate independently: membrane partitioning determines access, and electronic structure determines recognition within the accessible pool. The zero-shot transfer of the Pdr5p-trained classifier to human P-glycoprotein—without retraining, fine-tuning, or parameter adjustment—demonstrates that these constraints are not yeast-specific heuristics but reflect fundamental physical requirements imposed by the lipid bilayer environment. It must be emphasized that this transfer result should be interpreted as proof-of-concept evidence for conservation rather than a validated predictive tool for clinical application; the confidence interval for the internal accuracy estimate is wide [81.0%, 99.9%], and validation on additional transporters and larger datasets is required before generalization claims can be made. This conservation across ~1.5 billion years of evolutionary divergence suggests that membrane-gated electronic discrimination may be a universal organizing principle of polyspecific ABC transporter efflux. Practically, these findings provide a quantum chemical framework for early-stage efflux liability prediction. Compounds with LogP below the membrane-access threshold or with diffuse frontier orbitals are systematically poor Pdr5p substrates—a rule that generalizes to human ABCB1 and likely beyond. Expanding this framework to larger annotated datasets, explicit-membrane simulations, and additional transporter families represents the natural next step toward a general predictive model of multidrug resistance.

## Figures and Tables

**Table 1:** Electronic properties of Pdr5p substrates grouped by functional category. Values represent mean ± standard deviation. Gap = HOMO–LUMO gap; χ = electronegativity; η = chemical hardness.

Category	n	Gap (eV)	χ (eV)	η (eV)
Chemotherapy	5	3.70 ± 1.07	4.21 ± 0.54	1.85 ± 0.53
Membrane stress	6	4.35 ± 0.94	3.89 ± 0.72	2.18 ± 0.47
Antifungal	14	4.73 ± 0.59	3.52 ± 0.38	2.37 ± 0.30
Antibiotic	5	4.80 ± 0.53	3.68 ± 0.41	2.40 ± 0.27
Steroid	4	5.01 ± 0.39	3.61 ± 0.22	2.50 ± 0.20
Oxidative stress	12	5.09 ± 0.70	3.44 ± 0.55	2.54 ± 0.35
Control (non-substrate)	7	5.99 ± 1.17	2.93 ± 0.48	3.00 ± 0.58

**Table 2:** Frontier orbital localization and resistance by substrate category.

Category	n	HOMO Spread (%)	LUMO Spread (%)	Avg. Resistance (Fold)
Chemotherapy	5	1.83	2.10	3.7×
Membrane stress	6	2.11	2.08	9.3×
Antifungal	14	2.56	2.21	277× ^a^
Oxidative stress	12	1.79	1.80	2.3×
Control (non-substrate)	7	4.18	7.22	1.1×

Spread values represent the percentage of grid volume occupied by orbital density above threshold (|ψ| > 0.01 a.u.). ^a^ The high average resistance for the antifungal category (277×) is dominated by fluconazole (638-fold); median resistance across antifungals is 18×.

**Table 3:** Descriptor ablation study: SVM model performance across five descriptor sets evaluated by leave-one-out cross-validation. Classical descriptors: LogP, MW, TPSA, HBD, HBA, rotatable bonds. Quantum descriptors: HOMO, LUMO, gap, hardness, electronegativity, dipole. Combined: classical + quantum. Full: combined + Gap × LogP + TPSA/MW + HBD/MW. *p*-values from 500-shuffle permutation test.

Model	n	Accuracy (%)	Balanced Acc. (%)	Sensitivity (%)	Specificity (%)	MCC	*p*-Value
M0: LogP alone	61	83.6	90.7	81.5	100.0	0.579	0.0040
M1: Classical (6 descriptors)	61	82.0	77.4	83.3	71.4	0.415	0.0200
M2: Quantum (6 descriptors)	61	85.2	73.0	88.9	57.1	0.396	0.0319
M3: Combined (12 descriptors)	61	90.2	69.6	96.3	42.9	0.455	0.0379
M4: Full set (15 descriptors)	61	95.1	84.8	98.2	71.4	0.745	0.0020

**Table 4:** Classification performance of machine learning models evaluated by leave-one-out cross-validation.

Model	Accuracy	Sensitivity	Specificity	AUC-ROC	Balanced Accuracy	MCC
Logistic Regression	88.9%	100%	57.1%	0.86	78.5%	0.705
Random Forest	88.9%	100%	57.1%	0.84	78.5%	0.705
SVM (RBF)	92.6%	100%	71.4%	0.88	85.7%	0.806
k-Nearest Neighbors (k = 3)	92.6%	100%	57.1%	0.86	78.5%	0.705
Hybrid (SVM + rules)	96.3%	100%	85.7%	0.93	92.8%	0.904

**Table 5:** Threshold sensitivity of frontier orbital spread values by compound category.

Category	n	HOMO Spread (%)	LUMO Spread (%)				
		0.005	0.01 ^a^	0.02	0.005	0.01 ^a^	0.02
Chemotherapy	3	3.28	1.83	0.89	3.56	2.10	1.08
Membrane stress	2	3.72	2.06	0.98	3.03	1.70	0.85
Antifungal	3	4.45	2.56	1.28	4.13	2.21	1.10
Oxidative stress	3	3.24	1.79	0.88	3.44	1.80	0.84
Antibiotic	1	4.01	2.20	1.07	6.12	2.82	1.12
Control (non-substrate)	2	7.13	4.19	2.15	12.21	7.22	3.71

**^a^** Threshold used throughout the manuscript (0.01 a.u.). Values represent category means. The non-substrate > substrate ordering is preserved at all three thresholds for both HOMO and LUMO.

## Data Availability

The data presented in this study are available upon request from the corresponding author.

## Supplementary Materials

The following supporting information can be downloaded at https://www.mdpi.com/article/10.3390/molecules31122084/s1. Table S1. Comprehensive annotated dataset of Pdr5p substrates and non-substrates with supporting experimental evidence. Table S2. Descriptor ablation study showing SVM performance across five molecular descriptor sets evaluated by leave-one-out compound dataset. Table S3. Robustness analysis of the frontier orbital spread metric under grid-box perturbations for the 14-compound orbital analysis subset.

## Abbreviations

The following abbreviations are used in this manuscript:

ABC	ATP-binding cassette
MDR	multidrug resistance
PDR	pleiotropic drug resistance
DFT	density functional theory
FMO	frontier molecular orbital
HOMO	highest occupied molecular orbital
LUMO	lowest unoccupied molecular orbital
MEP	molecular electrostatic potential
PES	potential energy surface
SVM	support vector machine
LOOCV	leave-one-out cross-validation
ANOVA	analysis of variance
LogP	octanol–water partition coefficient
TPSA	topological polar surface area
HBD	hydrogen bond donor
HBA	hydrogen bond acceptor
AUC-ROC	area under the receiver operating characteristic curve
MCC	Matthews correlation coefficient
SAR	structure–activity relationship
