# Supplementary material for: Membrane Access and Orbital Localization Govern ABC Transporter Substrate Recognition
Source: Molecules. 2026 Jun 13;31(12):2084. doi: 10.3390/molecules31122084 (PMC13304817; doi:10.3390/molecules31122084)
Supplement: Supplementary file 1 [file molecules-31-02084-s001.zip › molecules-4342110-supplementary.pdf]

Table S1. Comprehensive annotated dataset of Pdr5p substrates and non-substrates with supporting experimental evidence.

Compound	Category	Label	Resistance(fold)	ResistanceNote	LogP	Assay Type	EvidenceQuality	In DFT(n=61)	In ML(n=61)	In OrbitalAnalysis (n=14)
Azithromycin	Antibiotic	SUBSTRATE	3.0	—	1.901	growth inhibition	Reported	✓	✓	✗
Chloramphenicol	Antibiotic	SUBSTRATE	4.0	—	0.909	drug accumulation; growth inhibition	Strong	✓	✓	✗
Ciprofloxacin	Antibiotic	SUBSTRATE	4.0	—	1.369	drug accumulation; growth inhibition	Moderate	✓	✓	✗
Erythromycin	Antibiotic	SUBSTRATE	3.0	—	1.786	growth inhibition	Moderate	✓	✓	✗
Norfloxacin	Antibiotic	SUBSTRATE	4.0	—	1.226	growth inhibition	Reported	✓	✓	✗
Tetracycline	Antibiotic	SUBSTRATE	3.0	—	-0.214	growth inhibition	Moderate	✗	✗	✗
Amorolfine	Antifungal	SUBSTRATE	5.0	—	5.068	growth inhibition	Moderate	✓	✓	✗
Ciclopirox	Antifungal	SUBSTRATE	4.0	—	1.474	growth inhibition	Reported	✓	✓	✗
Clotrimazole	Antifungal	SUBSTRATE	10.0	—	5.377	growth inhibition	Moderate	✓	✓	✗
Econazole	Antifungal	SUBSTRATE	6.0	—	5.148	growth inhibition	Reported	✓	✓	✗
Fluconazole	Antifungal	SUBSTRATE	638.0	from rhodamine 6G competition assay	0.736	drug accumulation; growth inhibition; ATPase stimulation	Strong	✓	✓	✓
Griseofulvin	Antifungal	SUBSTRATE	6.0	—	2.810	growth inhibition; ATPase stimulation	Moderate	✓	✓	✗
Itraconazole	Antifungal	SUBSTRATE	16.0	—	6.243	drug accumulation; growth inhibition	Strong	✓	✓	✓
Ketoconazole	Antifungal	SUBSTRATE	18.0	—	4.206	drug accumulation; growth inhibition	Strong	✓	✓	✓
Miconazole	Antifungal	SUBSTRATE	8.0	—	5.801	growth inhibition	Moderate	✓	✓	✗
Naftifine	Antifungal	SUBSTRATE	3.0	—	3.418	growth inhibition	Reported	✓	✓	✗
Posaconazole	Antifungal	SUBSTRATE	8.0	approximate	7.131	growth inhibition	Reported	✓	✓	✗
Ravuconazole	Antifungal	SUBSTRATE	8.0	approximate	2.944	growth inhibition	Reported	✓	✓	✗
Sulconazole	Antifungal	SUBSTRATE	5.0	—	6.518	growth inhibition	Reported	✓	✓	✗
Terbinafine	Antifungal	SUBSTRATE	4.0	—	3.418	growth inhibition; drug accumulation	Moderate	✓	✓	✗
Voriconazole	Antifungal	SUBSTRATE	12.0	—	2.177	growth inhibition; drug accumulation	Moderate	✗	✗	✗
Daunorubicin	Chemotherapy	SUBSTRATE	4.0	—	1.029	drug accumulation; ATPase stimulation	Strong	✓	✓	✓
Doxorubicin	Chemotherapy	SUBSTRATE	5.0	—	0.001	drug accumulation; ATPase stimulation; growth inhibition	Strong	✓	✓	✓
Etoposide	Chemotherapy	SUBSTRATE	3.0	—	-0.070	growth inhibition	Moderate	✓	✓	✗
Mitoxantrone	Chemotherapy	SUBSTRATE	3.0	—	-0.139	drug accumulation; growth inhibition	Strong	✓	✓	✓
Tamoxifen	Chemotherapy	SUBSTRATE	4.0	—	5.996	growth inhibition	Moderate	✓	✓	✗
Vincristine	Chemotherapy	SUBSTRATE	2.0	—	5.293	growth inhibition	Reported	✗	✗	✗
Alanine	Control	NON-SUBSTRATE	1.0	amino acid; standard negative control	-0.582	drug accumulation (negative control); growth inhibition (no effect)	Strong	✓	✓	✗
Amphotericin B	Control	NON-SUBSTRATE	1.0	different mechanism (membrane ergosterol binding)	-0.806	growth inhibition (no Pdr5p-mediated effect)	Strong	✓	✓	✗
Caffeine	Control	NON-SUBSTRATE	1.1	weak/no effect; LogP=0.06 (marginally above 0)	0.062	growth inhibition (no significant effect in pdr5-Delta)	Moderate	✓	✓	✓
Caspofungin	Control	NON-SUBSTRATE	1.0	echinocandin; targets glucan synthase not transport	-4.353	growth inhibition (not affected by PDR5 deletion)	Moderate	✓	✓	✗
Flucytosine	Control	NON-SUBSTRATE	1.0	DFT convergence failure	-0.956	growth inhibition (mechanism distinct from ABC efflux)	Moderate	✗	✗	✗
Glucose	Control	NON-SUBSTRATE	1.0	canonical negative control; no efflux detected	-3.221	drug accumulation (negative); growth inhibition (no effect)	Strong	✓	✓	✓
Glycine	Control	NON-SUBSTRATE	1.0	amino acid; standard negative control	-0.970	drug accumulation (negative control); growth inhibition (no effect)	Strong	✓	✓	✗
Sucrose	Control	NON-SUBSTRATE	1.0	sugar; standard osmotic/negative control	-5.396	growth inhibition (no effect); osmotic control	Moderate	✓	✓	✗
Benomyl	Fungicide	SUBSTRATE	3.0	benzimidazole; cross-resistance confirmed	2.572	growth inhibition	Moderate	✓	✓	✗
Fludioxonil	Fungicide	SUBSTRATE	4.0	phenylpyrrole	2.738	growth inhibition	Moderate	✓	✓	✗
Iprodione	Fungicide	SUBSTRATE	3.0	dicarboximide	2.880	growth inhibition	Reported	✓	✓	✗
Cerulenin	Membrane Stress	SUBSTRATE	5.0	—	2.514	growth inhibition	Reported	✓	✓	✗
Cycloheximide	Membrane Stress	SUBSTRATE	10.0	—	1.041	drug accumulation; growth inhibition	Strong	✓	✓	✓
Gramicidin S	Membrane Stress	SUBSTRATE	20.0	MW>1000 Da; excluded from DFT	0.766	growth inhibition; ATPase stimulation	Moderate	✓	✓	✗
Oligomycin A	Membrane Stress	SUBSTRATE	8.0	MW>1000 Da; excluded from DFT	9.196	growth inhibition; ATPase stimulation	Moderate	✓	✓	✗
Rhodamine 6G	Membrane Stress	SUBSTRATE	18.0	—	4.183	drug accumulation; fluorescence efflux assay	Strong	✓	✓	✓
Rhodamine B	Membrane Stress	SUBSTRATE	8.0	—	6.363	fluorescence efflux; growth inhibition	Moderate	✓	✓	✓
Valinomycin	Membrane Stress	SUBSTRATE	6.0	MW>1000 Da; excluded from DFT	0.926	growth inhibition	Reported	✗	✗	✗

Curcumin	Natural Product	SUBSTRATE	3.0	—	3.370	drug accumulation	Moderate	✓	✓	✗
Epigallocatechin	Natural Product	SUBSTRATE	2.0	EGCG; green tea catechin	1.252	growth inhibition	Reported	✓	✓	✗
Quercetin	Natural Product	SUBSTRATE	2.0	flavonoid	2.011	growth inhibition	Reported	✓	✓	✗
Resveratrol	Natural Product	SUBSTRATE	3.0	polyphenol; transport inferred from accumulation	2.974	drug accumulation; growth inhibition	Moderate	✓	✓	✗
4 Hydroxynonenal	Oxidative Stress	SUBSTRATE	2.0	reactive aldehyde	1.683	growth inhibition; electrophile efflux	Moderate	✓	✓	✗
Acrolein	Oxidative Stress	SUBSTRATE	2.0	—	0.371	growth inhibition	Reported	✓	✓	✗
Crotonaldehyde	Oxidative Stress	SUBSTRATE	2.0	—	0.761	growth inhibition	Reported	✓	✓	✗
Cystine	Oxidative Stress	SUBSTRATE	2.0	oxidised cysteine dimer	-0.808	efflux assay	Moderate	✓	✓	✗
Glutathione Oxidized	Oxidative Stress	SUBSTRATE	2.0	GSSG efflux	-3.877	GSSG efflux assay	Moderate	✓	✓	✓
Glutathione Reduced	Oxidative Stress	SUBSTRATE	3.0	GSH efflux quantified directly	-2.206	GSH efflux assay; drug accumulation	Strong	✓	✓	✓
Glyoxal	Oxidative Stress	SUBSTRATE	2.0	—	-0.616	growth inhibition	Reported	✓	✓	✗
Hexanal	Oxidative Stress	SUBSTRATE	2.0	—	1.766	growth inhibition	Reported	✓	✓	✗
Hydroxymethylfurfural	Oxidative Stress	SUBSTRATE	2.0	—	0.584	growth inhibition	Reported	✓	✓	✗
Malondialdehyde	Oxidative Stress	SUBSTRATE	2.0	—	0.257	growth inhibition	Reported	✓	✓	✗
Methylglyoxal	Oxidative Stress	SUBSTRATE	2.0	—	-0.226	growth inhibition; ATPase stimulation	Moderate	✓	✓	✗
S Nitrosoglutathione	Oxidative Stress	SUBSTRATE	2.0	GSNO; nitrosative stress	-1.721	GSNO efflux; growth inhibition	Moderate	✓	✓	✓
Corticosterone	Steroid	SUBSTRATE	4.0	—	1.782	ATPase stimulation	Moderate	✓	✓	✗
Cortisol	Steroid	SUBSTRATE	3.0	—	1.782	ATPase stimulation	Reported	✓	✓	✗
Estradiol	Steroid	SUBSTRATE	3.0	—	3.609	ATPase stimulation; growth inhibition	Moderate	✓	✓	✗
Progesterone	Steroid	SUBSTRATE	5.0	—	4.724	ATPase stimulation; drug accumulation	Strong	✓	✓	✗
Testosterone	Steroid	SUBSTRATE	4.0	—	3.879	ATPase stimulation	Moderate	✓	✓	✗

Table S2. Descriptor ablation study showing SVM performance across five molecular descriptor sets evalu
compound dataset.

Model	n	Accuracy(%)	alancedAccuracy (%)	Sensitivity(%)	Specificity(%)
M0: LogP alone	61	83.6	90.7	81.5	100.0
M1: Classical 6	61	82.0	77.4	83.3	71.4
M2: Quantum 6	61	85.2	73.0	88.9	57.1
M3: Combined 12	61	90.2	69.6	96.3	42.9
M4: Full set 15	61	95.1	84.8	98.2	71.4

ated by leave-one-out cross-validation (LOOCV) on the 61-

MCC	TP	TN	FP+FN	p-value (permutation)
0.579	44	7	10	0.0040
0.415	45	5	11	0.0200
0.396	48	4	9	0.0319
0.455	52	3	6	0.0379
0.745	53	5	3	0.0020

Table S3. Robustness analysis of the frontier orbital spread metric under grid-box perturbations for the 14-compound orbital analysis subset.

Compound	Non-sub	Original0.01 a.u.	Crop 10%0.01 a.u.	Crop 20%0.01 a.u.	Pad 20%0.01 a.u.	Subsample2× 0.01 a.u.	LUMOOriginal	LUMOCrop 10%	LUMOCrop 20%	LUMOPad 20%
Caffeine	Yes	4.18	8.16	19.15	1.52	4.18	4.51	8.81	20.89	1.64
Cycloheximide	Yes	2.20	4.29	10.15	0.80	2.21	2.82	5.51	13.05	1.03
Daunorubicin	Yes	1.78	3.48	8.24	0.65	1.76	2.12	4.14	9.60	0.77
Doxorubicin	Yes	1.74	3.39	8.03	0.63	1.74	2.02	3.94	9.22	0.73
Fluconazole	Yes	3.74	7.31	16.93	1.36	3.76	3.14	6.14	14.18	1.15
Glucose	Yes	4.19	8.19	18.84	1.53	4.18	7.22	13.95	26.39	2.63
Glutathione Oxidized	Yes	1.43	2.79	6.61	0.52	1.43	1.22	2.37	5.62	0.44
Glutathione Reduced	Yes	1.93	3.75	6.84	0.70	1.95	2.84	5.55	10.58	1.04
Itraconazole	Yes	1.91	3.73	8.81	0.70	1.91	1.42	2.68	2.90	0.52
Ketoconazole	Yes	2.03	3.97	9.41	0.74	2.05	2.07	4.04	9.57	0.75
Mitoxantrone	Yes	1.98	3.87	9.16	0.72	1.99	2.17	4.25	9.69	0.79
Rhodamine 6G	Yes	1.90	3.71	8.66	0.69	1.90	1.76	3.44	8.12	0.64
Rhodamine B	Yes	2.22	4.33	9.99	0.81	2.20	1.65	3.22	7.53	0.60
S Nitrosoglutathione	Yes	2.02	3.94	8.84	0.74	2.01	1.36	2.65	5.57	0.49
Non-substrate mean	—	nan	nan	nan	nan	nan	nan	nan	nan	nan
